# Effect of dietary fat on hepatic liver X receptor expression in P-glycoprotein deficient mice: implications for cholesterol metabolism

**DOI:** 10.1186/1476-511X-7-21

**Published:** 2008-06-11

**Authors:** Sheila J Thornton, Evelyn Wong, Stephen D Lee, Kishor M Wasan

**Affiliations:** 1 University of British Columbia, Faculty of Pharmaceutical Sciences, 2146 University Blvd, Vancouver, British Columbia, V6T 1Z3, Canada

## Abstract

Pgp (P-glycoprotein, MDR1, ABCB1) is an energy-dependent drug efflux pump that is a member of the ATP-binding cassette (ABC) family of proteins. Preliminary studies have reported that nonspecific inhibitors of Pgp affect synthesis and esterification of cholesterol, putatively by blocking trafficking of cholesterol from the plasma membrane to the endoplasmic reticulum, and that relative increases in Pgp within a given cell type are associated with increased accumulation of cholesterol. Several key efflux proteins involved in the cholesterol metabolic pathway are transcriptionally regulated by the nuclear hormone liver X receptor (LXR). Therefore, to examine the interplay between P-glycoprotein and the cholesterol metabolic pathway, we utilized a high fat, normal cholesterol diet to upregulate LXRα without affecting dietary cholesterol. Our research has demonstrated that mice lacking in P-glycoprotein do not exhibit alterations in hepatic total cholesterol storage, circulating plasma total cholesterol levels, or total cholesterol concentration in the bile when compared to control animals on either a normal (25% calories from dietary fat) or high fat (45% calories from dietary fat) diet. However, p-glycoprotein deficient mice (*Mdr1a*^-/-^/*1b*^-/-^) exhibit increased hepatic LXRα protein expression and an elevation in fecal cholesterol concentration when compared to controls.

## Background

Cholesterol homeostasis of an organism involves the interplay of two main input mechanisms (hepatic and extra-hepatic synthesis of cholesterol, and the intestinal absorption of dietary and biliary cholesterol), balanced with a single elimination pathway via the bile (either through direct excretion into the bile or by conversion to bile acids). Other pathways such as steroidogenesis, myelination and anabolic growth play a minor role in the daily cholesterol balance. Physiologically, an organism has a large but not unlimited capacity to accommodate increases in dietary uptake. When dietary cholesterol levels exceed the capacity of efflux mechanisms and override compensatory decreases in biosynthesis, an imbalance in cholesterol occurs and excessive accumulation ensues.

Several key efflux proteins involved in the cholesterol metabolic pathway are transcriptionally regulated by the nuclear hormone liver X receptor (LXR). When stimulated by oxysterols, LXR forms an obligate heterodimer with retinoid X receptor (RXR) and binds to specific DNA response elements, inducing the expression of genes involved in cholesterol efflux pathways [1]. The LXR/RXR dimer can be activated by ligands for either receptor, resulting in a complex interplay between dietary fat and cholesterol induction of the nuclear receptor response [2]. LXRα is highly expressed in the liver, and is also found in adipose, intestine, kidney and macrophages, while LXRβ is ubiquitous.

Pgp (P-glycoprotein, MDR1, ABCB1) is an energy-dependent drug efflux pump that is a member of the ATP-binding cassette (ABC) family of proteins [3]. Preliminary studies have reported that nonspecific inhibitors of Pgp inhibit synthesis and esterification of cholesterol, putatively by blocking trafficking of cholesterol from the plasma membrane to the endoplasmic reticulum [4,5], and that relative increases in Pgp within a given cell type are associated with increased accumulation of cholesterol [6,7]. These findings provide indirect evidence supporting a physiologic function for Pgp in the homeostasis of cholesterol. At the whole animal level, a Pgp deficient mouse model (*Mdr1a*^-/-^/*1b*^-/-^) exhibited a decrease in hepatic cholesterol accumulation and enhanced esterification when compared to control mice. However, these animals did not appear to display any change in dietary cholesterol absorption [8].

As much of the evidence surrounding the Pgp and cholesterol interaction is correlative, our analysis was undertaken to establish a specific interface between Pgp function and the cholesterol metabolic pathway. Using a p-glycoprotein knockout mouse model (*Mdr1a*^-/-^/*1b*^-/-^), we examined the effect of Pgp deficiency on the plasma cholesterol, hepatic cholesterol storage and cholesterol excretion. We then evaluated hepatic LXRα expression and downstream targets to shed light on the complex interaction between this membrane-bound protein and cholesterol metabolic pathways. Using dietary fat as a natural LXR ligand, we assessed hepatic LXRα expression in animals fed cholesterol-controlled diets (0.02%) corresponding to normal dietary fat content (NF; 25% calories from lipids) and high dietary fat content (HF; 45% calories from lipids).

## Methodology

Male P-glycoprotein knockout mice in an FVB background (FVB.129P2-*Abcb1a*^*tm*1*Bor*^*Abcb1b*^*tm*1*Bor *^N12) and FVB control mice were obtained from Taconic Farms Inc. and maintained for 12 weeks on either a normal fat (NFNC; 25% of calories from fat; n = 8 per genotype) or high fat diet (HFNC; 45% of calories from fat; n = 8 per genotype) containing 0.02% cholesterol (Research Diets). All animal protocols were approved by the University of British Columbia's Animal Care Committee and conform to the Canadian Council on Animal Care guidelines.

### Total cholesterol

Fecal lipids were extracted using a Folch extraction protocol [9]. Briefly, approximately 100 mg of feces was homogenized for 2 min in a 2:1 CHCl_3_:CH_3_OH solution (20:1 chloroform/methanol to fecal mass).

Homogenates were oscillated overnight at room temperature and then filtered through glass wool. Filtrate was washed with 0.9% saline and centrifuged at 1000 g and 4°C for 10 minutes. The upper phase was removed and the interface washed three times with pure upper phase. The lower phase was then dried under nitrogen gas. Lipid films were reconstituted in 300 μl of 10% Triton-X in isopropanol. Total fecal cholesterol levels were then determined using the Wako Cholesterol E assay kit.

### PLTP activity

Plasma PLTP activity was determined via the transfer of a fluorescent substrate from donor to acceptor (PLTP activity assay kit, Roar Biomedicals). Plasma total cholesterol levels were determined using an enzymatic colorimetric assay (Cholesterol E kit, Wako Chemicals).

### Western blotting (hepatic LXR expression)

Liver samples weighing 30–50 mg were homogenized in 700 μl of RIPA buffer containing 1 μM PMSF and 1% Protein Inhibitor Cocktail (Sigma). The samples were centrifuged at 14 000 rpm and 4°C for 15 minutes and the supernatant was obtained. Protein concentration in each sample was determined using the bicinchoninic acid assay (BCA) (Pierce).

For LXRα protein expression, 130 μg of protein was run on a 1.5 mm thick 12.5%T SDS-polyacrylamide gel and transferred onto a nitrocellulose membrane (Bio-Rad). The membrane was blocked with 5% skim milk in Tris-buffered saline containing 0.1% Tween-20 (TBS-T) for 2 hours at room temperature, cut at the 43 kDa mark, and incubated in primary antibody overnight at 4°C (1:500 anti-LXRα (Abcam) and 1:300 anti-Histone H1 (Santa Cruz Biotech)). The secondary antibodies were incubated for 1 hour at 4°C (1:3000 goat anti-rabbit and 1:3000 goat anti-mouse IgG_2A _antibody (Santa Cruz Biotech) respectively).

The membranes were then exposed to ECL western blotting detection reagents (Amersham Biosciences), and band density was determined. Data are expressed as the ratio of LXRα to histone protein expression.

### Statistical analyses

Statistical analyses were conducted using JMP software V 5.1 for Windows (SAS Institute Inc, Carey, NC, USA). A one-way ANOVA was used to establish significant differences between groups, and different group means were then separated by a Tukey-Kramer test for Honestly Significant Difference (HSD); differences were considered statistically significant when P < 0.05.

## Results

### Total cholesterol

**Figure 1:**
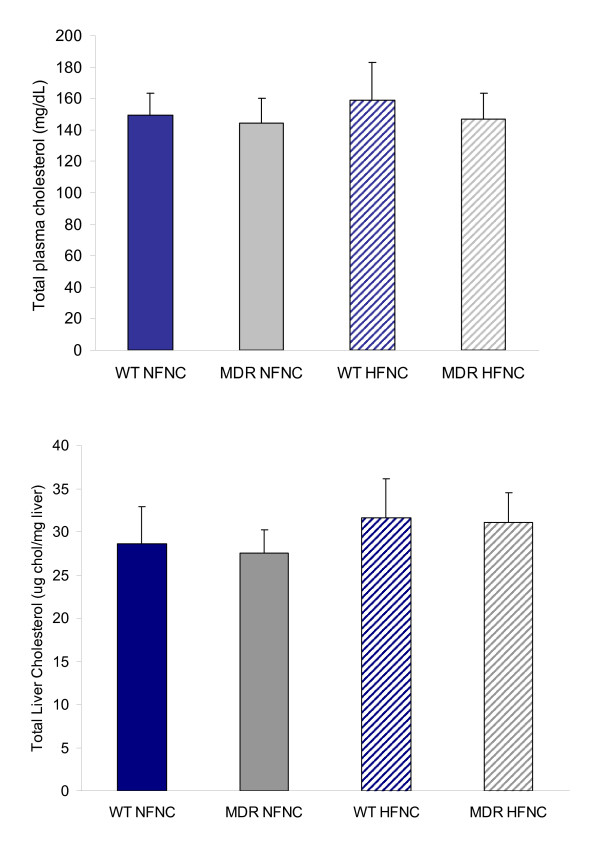
Total cholesterol storage in plasma (1a) and liver (1b) collected from control and P-glycoprotein deficient mice (*Mdr1a/*^-/-^/*1b*^-/-^) fed normal fat and high fat diets (*Mdr1a/*^-/-^/*1b*^-/- ^genotype is designated "MDR" in all graphs; FVB wild type mouse is designated "WT"). No significant differences in total cholesterol in the liver or plasma were observed (n = 8; p > 0.05).

**Figure 2:**
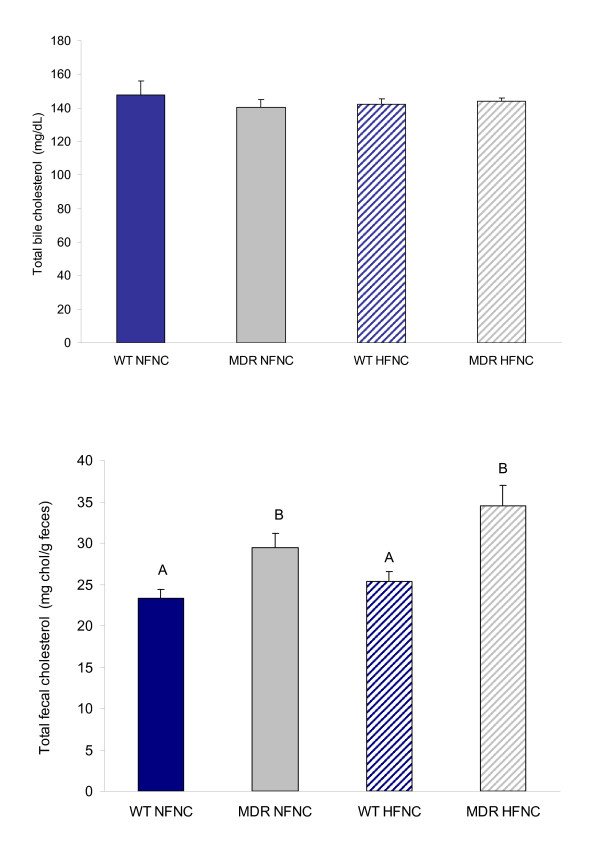
Total cholesterol efflux from bile (2a) and feces (2b) collected from control and P-glycoprotein deficient mice (*Mdr1a/*^-/-^/*1b*^-/-^) fed normal fat and high fat diets (*Mdr1a/*^-/-^/*1b*^-/- ^genotype is designated "MDR" in all graphs; FVB wild type mouse is designated "WT"). No significant differences in total cholesterol in the bile were observed (n = 8; p > 0.05). However, the *Mdr1a/*^-/-^/*1b*^-/- ^genotype was associated with a significant increase in fecal cholesterol concentration (n = 7; p < 0.05). Differing letter designations indicate statistically significant data.

*Mdr1a*^-/-^/*1b*^-/- ^animals on either a NF or HF diet did not exhibit any differences in plasma total cholesterol, hepatic cholesterol storage, or in the concentration of total cholesterol excreted in the bile when compared to wild type (Fig. 1a, b and Fig. 2a). However, a significant difference in fecal total cholesterol levels was observed when comparing *Mdr1a*^-/-^/*1b*^-/- ^animals to wild type (Fig. 2b).

### Hepatic LXR protein expression

**Figure 3:**
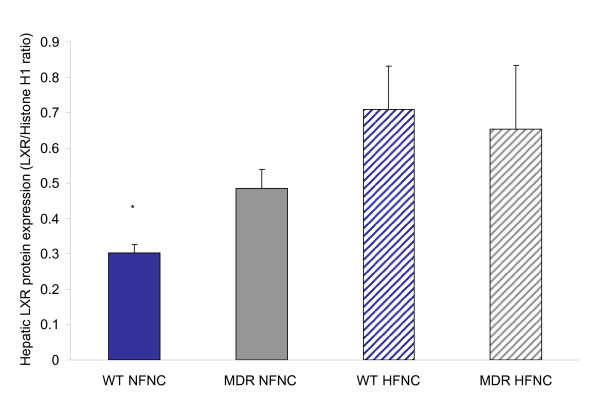
Hepatic LXRa protein expression in P-glycoprotein deficient mice (*Mdr1a/*^-/-^/*1b*^-/-^) fed normal and high fat diets. A significant increase in protein expression with both the *Mdr1a/*^-/-^/*1b*^-/- ^genotype and with a high fat diet when compared to wild type animals on a normal fat diet (n = 7; p < 0.05 as noted by asterisk). Placing an *Mdr1a/*^-/-^/*1b*^-/-^animal on a high fat diet did not result in further upregulation of protein expression.

A significant increase in liver LXRα protein expression was found in the HF WT group compared to the NF WT (Fig. 3). There was no significant difference in liver LXRα protein expression when comparing diets in *Mdr1a*^-/-^/*1b*^-/- ^animals; however, there was a general trend for increased liver LXRα protein expression. A significant increase in liver LXRα protein expression was found in the *Mdr1a*^-/-^/*1b*^-/- ^group compared to the WT group in mice fed a NF diet. There was no significant difference in liver LXRα protein expression when comparing *Mdr1a*^-/-^/*1b*^-/- ^to WT mice fed a HF diet. No significant difference was seen in the HF diet in LXRα expression.

### Plasma PLTP activity

**Figure 4:**
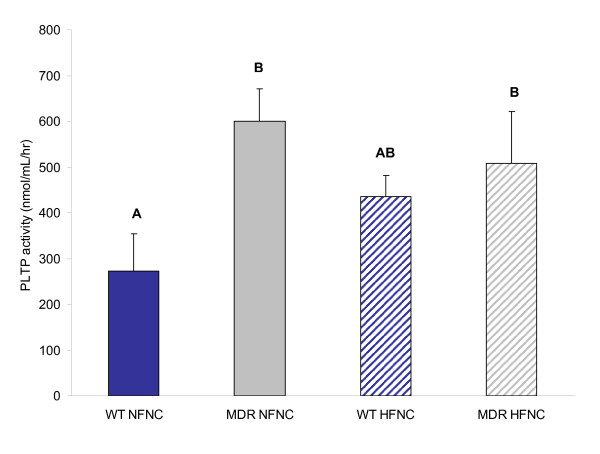
Plasma phospholipid transfer protein (PLTP) activity in P-glycoprotein deficient mice (*Mdr1a/*^-/-^/*1b*^-/-^) fed normal and high fat diets. A significant increase in PLTP activity was associated with the P-glycoprotein deficient genotype (n = 5; p < 0.05). Differing letter designations indicate statistically significant data.

There was no significant difference in PLTP activity when comparing NF and HF diets; however, there was a general trend for increased PLTP activity in the WT group fed a HF diet (Fig. 4). A significant increase in PLTP activity was seen in *Mdr1a*^-/-^/*1b*^-/- ^mice compared to WT mice fed a NF diet. These findings of increased PLTP activity, contributing to cholesterol clearance, and elevation of plasma HDL, are consistent with the increases in liver LXRα protein expression.

### Hepatic ABCG5 protein expression

**Figure 5:**
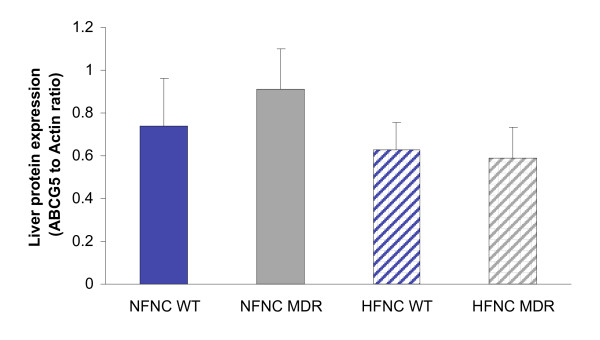
Hepatic ABCG5 protein expression in P-glycoprotein deficient mice (*Mdr1a/*^-/-^/*1b*^-/-^) fed normal and high fat diets. No significant differences in ABCG5 levels were observed with change in dietary fat or with genotype (n = 5; p > 0.05).

In the mouse liver, the level of ABCG5 cholesterol efflux protein did not show a significant upregulation with genotype or diet (Fig. 5).

## Discussion

A connection between the integral membrane ABC transporter P-glycoprotein and the cholesterol metabolic pathway has been proposed [10]. *In vitro*, nonspecific inhibitors of Pgp inhibit cholesterol esterification and trafficking [5-7], while *in vivo *research suggests hepatic accumulation and esterification are enhanced in *Mdr1a*^-/-^/*1b*^-/- ^mice [8]. Our research has demonstrated that mice lacking in P-glycoprotein do not exhibit alterations in hepatic total cholesterol storage, circulating plasma total cholesterol levels, or total cholesterol concentration in the bile when compared to control animals on either a normal (25% calories from dietary fat) or high fat (45% calories from dietary fat) diet. However, *Mdr1a*^-/-^/*1b*^-/- ^mice exhibit increased hepatic LXRα protein expression and an elevation in fecal cholesterol concentration when compared to controls.

LXRs are "cholesterol sensors" which, in response to excess cholesterol, stimulate its transport to the liver and facilitate biliary excretion. Various saturated and unsaturated fatty acids are also observed to increase LXRα expression in cultured rat hepatoma cells[11]. In the rat, high-fat diet and fasting are associated with an up-regulation of hepatic LXRa expression, likely through the elevation of the plasma fatty acid levels [12]. To examine the interplay between P-glycoprotein and the cholesterol metabolic pathway, we utilized a high fat, normal cholesterol diet to upregulate LXRα without affecting dietary cholesterol. Our data indicate that administration of a chronic HF diet to mice results in a significant upregulation of LXRα protein expression when compared to the NF diet wild type mice. In addition, P-glycoprotein deficient mice fed a normal chow diet exhibit an equivalent statistically significant increase in LXRα expression when compared to wild type mice. When P-glycoprotein deficient mice are placed on a chronic HF diet, no further upregulation in LXRα expression is observed (Fig 3).

In human macrophages, adipocytes, and hepatocytes [13], LXRα expression is controlled by an auto-regulatory mechanism. The human LXRα gene promoter contains three functional LXR response elements (LXREs), one of which is strongly activated by both LXRα and LXRβ[14]. Although the amount of LXRα mRNA in white mouse adipose tissue increases in response to the synthetic LXR agonist T0901317, murine LXRα is not autoregulated in other cell types, such as macrophages and hepatocytes [15]. Given the available data, we cannot identify the specific modulator that results in upregulation of hepatic LXRα protein expression in the *Mdr1a*^-/-^/*1b*^-/- ^mouse model; however, the data strongly support the P-glycoprotein/cholesterol relationship by demonstrating a significant upregulation of a top-level cholesterol sensing control mechanism, strengthening the link between P-glycoprotein and the cholesterol metabolic pathway.

To confirm if a rise in hepatic LXRα protein expression translates to increased LXR activity and upregulation of downstream targets, we evaluated the activity of plasma phospholipid transfer protein (PLTP), a known direct target gene of LXRα. In mice, disruption of PLTP function dramatically reduces plasma HDL cholesterol concentration [16]. Treatment with LXRα agonists is known to result in elevated circulating HDL levels, and it has been suggested that upregulation of PLTP may be responsible [17]. Our findings demonstrate that the *Mdr1a*^-/-^/*1b*^-/- ^mouse model exhibits greater plasma PLTP activity when compared to wild type mice. A trend toward increased PLTP activity with high fat diet is also observed, with activity results closely following hepatic LXRα protein expression data.

As LXR is known to upregulate transcription of proteins involved in cholesterol efflux pathways, our finding of increased LXR expression in the *Mdr1a*^-/-^/*1b*^-/- ^mouse model when compared to wild type is congruent with the observed increase in fecal total cholesterol content (Fig 2b). However, this fecal cholesterol loss may be due to one or all of the following: increased cholesterol efflux from the liver via the bile (mediated by the heterodimeric pair of proteins ABCG5 and 8; [18]); an increase in cholesterol efflux from the enterocyte into the intestinal lumen via ABCA1 [19] or intestinal ABCG5/8 [20]; or a decrease in cholesterol uptake from the intestinal lumen by a yet-unknown protein-facilitated mechanism.

To examine the potential role of cholesterol efflux from the liver as a contributor to the elevated fecal cholesterol, we quantified hepatic ABCG5 protein expression (Fig. 5) and found no significant difference in protein expression with genotype or diet. As ABCG5/8 heterodimer is the transporter responsible for cholesterol excretion from the liver into the bile, these data are in agreement with our findings of no significant differences in liver cholesterol storage or bile cholesterol concentration. However, we did not measure the rate of bile formation, therefore cannot rule out an increased hepatic cholesterol efflux at this time (although a significant increase in cholesterol efflux via this pathway is not likely, as the protein expression of ABCG5 did not increase in either the *Mdr1a*^-/-^/*1b*^-/- ^mouse model or with a chronic HF diet).

The second possible cause of elevated fecal cholesterol in the *Mdr1a*^-/-^/*1b*^-/- ^mouse model is an increase in cholesterol efflux from the enterocyte into the lumen. It has been demonstrated that activation of the RXR/LXR heterodimer by either a retinoid or LXR agonist effectively blocks cholesterol absorption [20].

Absorption of cholesterol is a three-step process and requires uptake across the luminal membrane followed by cytosolic transport and assembly with lipoproteins, culminating in the subsequent release of cholesterol into lymphatic or systemic circulation. The interruption of any one step along this trafficking route with compromise the absorptive process. Repa *et al *(2000) attribute the RXR/LXR heterodimer-induced decrease in cholesterol absorption to an observed increase in expression of intestinal ABCA1, a gene product responsible for efflux of cellular free cholesterol [19]. A subsequent study revealed that LXR induced upregulation in ABCG5 and G8 protein expression also contributes to decreased cholesterol absorption, suggesting that all three proteins play a role in decreasing cholesterol absorption via increased cholesterol efflux into the intestinal lumen [20].

A third mechanism that may be responsible for the observed elevation in fecal cholesterol concentration is the decreased uptake of dietary and biliary cholesterol. A number of findings support the presence of a protein-facilitated cholesterol uptake mechanism located at the enterocyte brush border. Firstly, cholesterol uptake appears to be sensitive to protease treatment and is a saturable process [21]. In addition, sterol uptake is highly selective, with significantly greater uptake of cholesterol occurring across the brush border membrane when compared to similar phytosterols [22,23]. The observed variation in the cholesterol absorption phenotype in various mouse strains, and the development of a specific cholesterol inhibitor, ezetimibe, are strongly suggestive of a genetic component in the cholesterol absorption process. However, the identity of a specific protein-facilitated cholesterol uptake mechanism has been highly controversial [24-28].

Given the indirect relationship between P-glycoprotein protein expression, activity, and cholesterol uptake, the direct transport of cholesterol across the plasma membrane by Pgp is unlikely. However, we cannot rule out Pgp as a contributor to this process. Pgp has been identified as a functional phospholipid and glycosphingolipid flippase, and is associated with cholesterol microdomains in cell membranes [29-32]. Based on the identification of the cholesterol binding site sequence from a benzodiazepine receptor protein [33], we have conducted a bioinformatics analysis to identify four putative cholesterol binding sites on the cytosolic portion of the P-glycoprotein molecule (Leon et al, in submission). The cytosolic location of these cholesterol binding sites on the p-glycoprotein molecule, combined with the evidence supporting an indirect relationship between P-glycoprotein and cholesterol metabolism, suggest that Pgp may act as a cholesterol "dock" which facilitates the transfer of sterol molecules to cytosolic transport proteins, such as Niemann-Pick C1-like1 (NPC1L1). The cytosolic cholesterol binding sites may facilitate the movement of cholesterol from the exofacial to cytofacial leaflet, assisting in the maintenance of membrane cholesterol asymmetry and supplementing the luminal/exofacial leaflet cholesterol gradient, thus facilitating cholesterol diffusion across the membrane. As P-glycoprotein acts as an efflux transporter and is found in the luminal surface of epithelial cells, it has the potential to assist in both cholesterol uptake across the enterocyte, as well as facilitate cholesterol efflux in the liver [29].

## Conclusion

The establishment of a direct link between P-glycoprotein and cholesterol metabolic pathway remains elusive. However, the data presented here further substantiate the connection between this membrane-bound protein and cholesterol transport. We have observed a significant increase in cholesterol efflux in P-glycoprotein deficient animals and established that the lack of P-glycoprotein results in alterations in the top level of the cholesterol metabolic pathway via increased expression of LXRα, a nuclear hormone crucial in orchestrating the complex processes involved in the trafficking of cholesterol. We have further postulated a potential mechanism by which P-glycoprotein may facilitate cholesterol transport across the membrane and, depending on the location of expression, supplement both uptake and efflux of sterol molecules.

## Authors' contributions

All authors have read and approve of the final manuscript. SJT designed the study, carried out the plasma cholesterol and PLTP assays, EW carried out the Western blot analyses for LXR and ABCG5 and conducted fecal lipid extraction and fecal cholesterol assays, SDL was responsible for animal husbandry, tissue collection and participated in the design of the study and interpretation of results, KMW participated in study coordination. This work was supported by Canadian Institutes of Health Research (CIHR) grant MOP-86447 to KMW and SJT.
